# Supplementary material for: Mathematical modeling to inform vaccination strategies and testing approaches for COVID-19 in nursing homes
Source: medRxiv. 2021 Mar 1:2021.02.26.21252483. Preprint. [Version 1] doi: 10.1101/2021.02.26.21252483 (PMC7941643; doi:10.1101/2021.02.26.21252483)
Supplement: 1 [file NIHPP2021.02.26.21252483-supplement-1.pdf]

medRxiv preprint doi: https://doi.org/10.1101/2021.02.26.21252483; this version posted March 1, 2021. The copyright holder for this preprint (which was not certified by peer review) is the author/funder, who has granted medRxiv a license to display the preprint in perpetuity. This article is a US Government work. It is not subject to copyright under 17 USC 105 and is also made available for use under a CC0 license.

Supplementary Materials

Supplemental Figure 1.

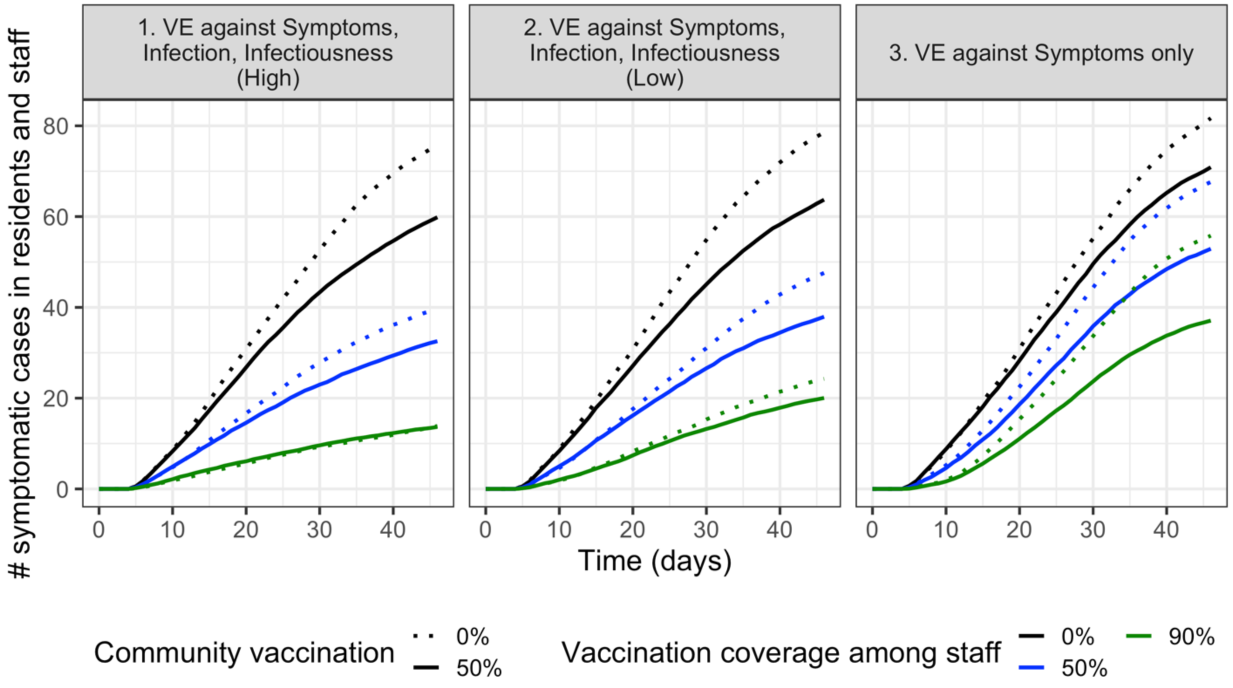

Figure S1. Dotted lines show original simulations from Figure 1 with no vaccination in the community. Solid lines show means of simulations done with 50% of incoming residents already vaccinated.

Supplemental Figure 2.

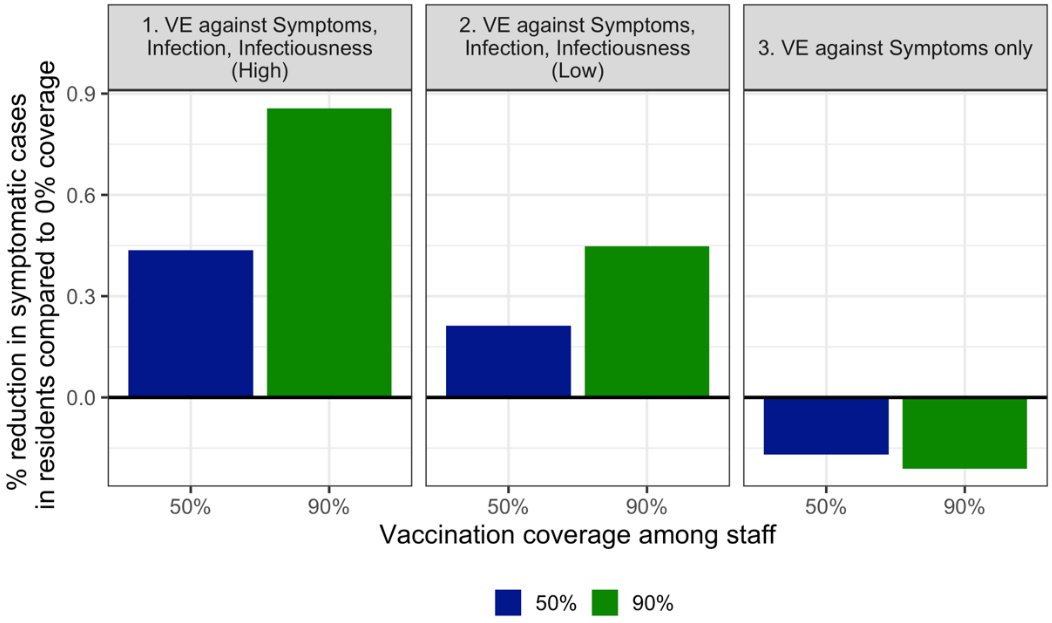

Figure S2. Effect of staff vaccination when 50% of incoming residents are vaccinated. When considering symptomatic cases in residents, increasing vaccination coverage among staff from 0% reduces cases in

medRxiv preprint doi: https://doi.org/10.1101/2021.02.26.21252483; this version posted March 1, 2021. The copyright holder for this preprint (which was not certified by peer review) is the author/funder, who has granted medRxiv a license to display the preprint in perpetuity.
This article is a US Government work. It is not subject to copyright under 17 USC 105 and is also made available for use under a CC0 license.

vaccine scenarios 1 and 2, when vaccination confers at least low protection against infection and infectiousness. When the vaccine protects against symptoms only, increasing vaccination coverage among staff increases symptomatic cases among residents.

Supplemental Figure 3.

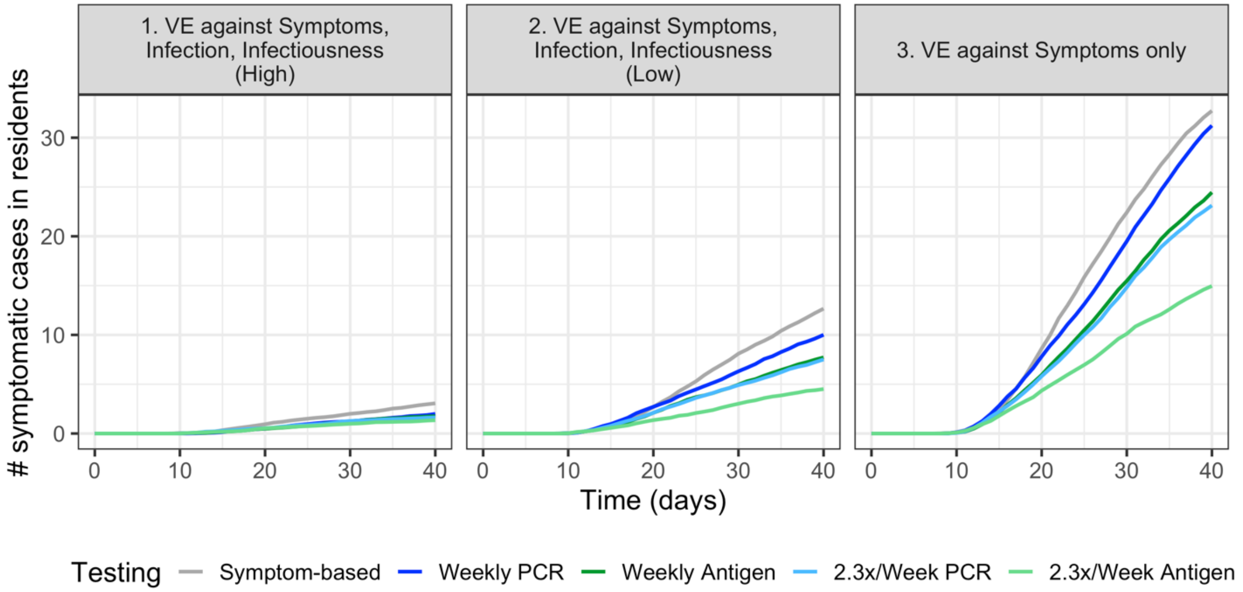

Figure S3. Effect of testing when 50% of incoming residents are vaccinated. When the vaccine has low or no efficacy against infections and infectiousness (Scenarios 2 and 3), frequent screening testing is important for reducing total symptomatic cases in residents. Due to faster turnaround time, antigen testing results in lower incidence than PCR testing at the same frequency.

Supplemental Figure 4.

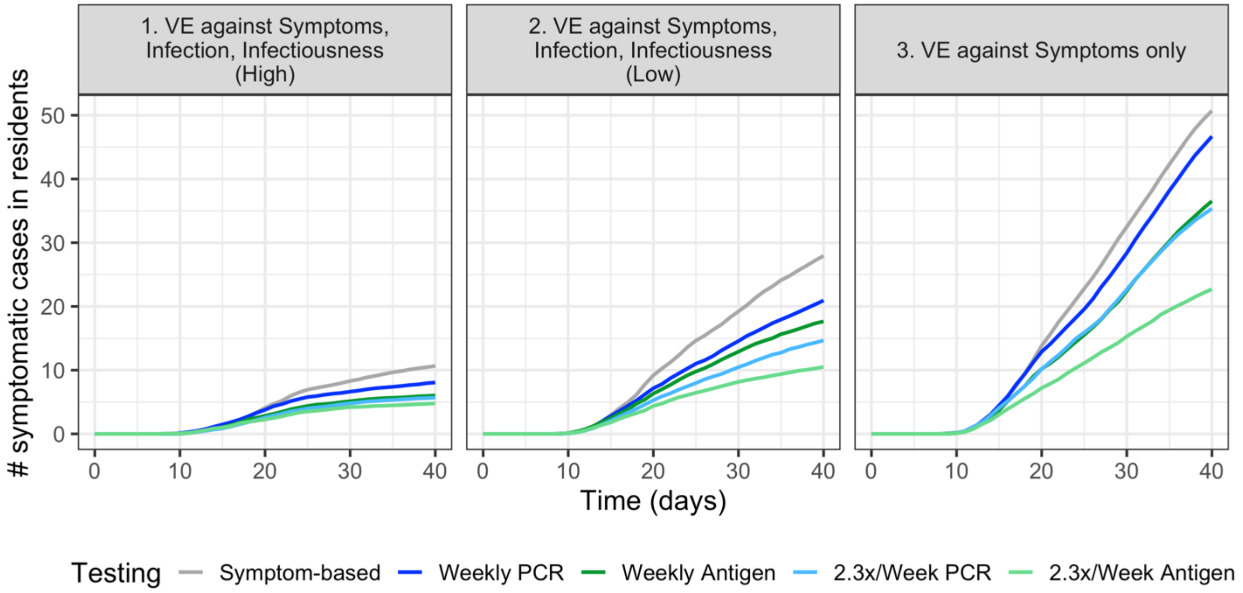

medRxiv preprint doi: https://doi.org/10.1101/2021.02.26.21252483; this version posted March 1, 2021. The copyright holder for this preprint (which was not certified by peer review) is the author/funder, who has granted medRxiv a license to display the preprint in perpetuity. This article is a US Government work. It is not subject to copyright under 17 USC 105 and is also made available for use under a CC0 license.

Figure S4. When cases are allowed to be introduced 8 days after the first vaccine dose (vs. 8 days after the second dose in Figure 3), we see that testing remains more important for controlling outbreak size under all VE scenarios. Here, the second dose is administered on day 13, and takes effect on day 20.

Table S1. Model parameters

**Parameter**	**Values***
Number of residents	100 [19]
Number of staff	100 [19]
Probability of infection per infectious contact	0.02
Latent period (days)	3-5 [25]
Time in infectious compartment (days); infectiousness dependent on viral load	14 [6]
Daily probability of infection from the community	0.03
Daily contacts staff-staff	2 [19]
Daily contacts residents - staff	6 [19,20]
Daily contacts staff - residents	6 - 12 [19]
Daily contacts residents - residents (non-roommates)	0 [19]
Proportion of staff asymptomatic	0.4 [26,27]
Proportion of residents asymptomatic	0.2 [28–30]
Duration of presymptomatic transmission (days)	2 [25,27,31]
Reduction in force of infection per contact from PPE	95% [32]
Proportion of temporary staff previously infected (and assumed immune) upon entry into nursing home	0.2
Proportion of incoming residents vaccinated	0, 50%
Baseline mortality (daily)	1/1000
COVID-19 mortality (daily)	2/100
Mean peak viral load (copies/mL)	10^8 [33]
Limit of detection - rapid antigen test (copies/mL)	10^5 [22, 34–36]
Limit of detection - PCR (copies/mL)	10^3 [22, 37]

medRxiv preprint doi: https://doi.org/10.1101/2021.02.26.21252483; this version posted March 1, 2021. The copyright holder for this preprint
(which was not certified by peer review) is the author/funder, who has granted medRxiv a license to display the preprint in perpetuity.
This article is a US Government work. It is not subject to copyright under 17 USC 105 and is also made available
for use under a CC0 license.

Antigen and PCR test specificity	1
Viral load threshold for infectiousness (copies/mL)	10^4
Viral load threshold for high infectiousness (copies/mL)	10^7
Turnaround time - antigen test (days)	Same day
Turnaround time - PCR test (days)	2
Time between vaccine dose 1 and 2 (days)	21 [9]
Time until effect of vaccine dose after vaccination (days)	7
Length of stay 25th, 50th, 75th percentiles, after restricting to minimum of 7 days (days)	15, 27, 66 [18,38]

*If no reference is cited, parameters are assumed.
